# Supplementary material for: Causal Relationship between Aging and Anorexia Nervosa: A White-Matter-Microstructure-Mediated Mendelian Randomization Analysis
Source: Biomedicines. 2024 Aug 16;12(8):1874. doi: 10.3390/biomedicines12081874 (PMC11351342; doi:10.3390/biomedicines12081874)
Supplement: Supplementary file 1 [file biomedicines-12-01874-s001.zip › Supplementary Figure/Figure S4/Singe SNP plot of MR analysis for TL to ubm-b-1695.pdf]

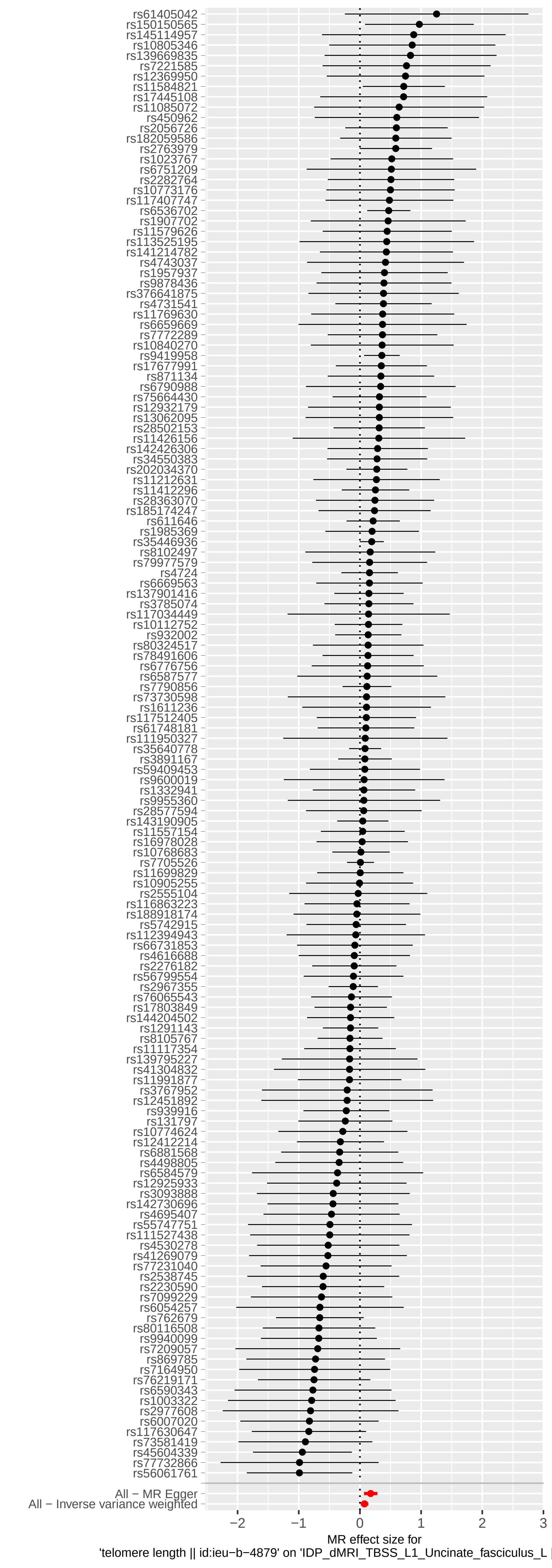

rs61405042
rs150150565
rs145114957
rs10805346
rs139669835
rs7221585
rs12369950
rs11584821
rs17445108
rs11085072
rs450962
rs2056726
rs182059586
rs2763979
rs1023767
rs6751209
rs2282764
rs10773176
rs117407747
rs6536702
rs1907702
rs11579626
rs113525195
rs141214782
rs4743037
rs1957937
rs9878436
rs376641875
rs4731541
rs11769630
rs6659669
rs7772289
rs10840270
rs9419958
rs17677991
rs871134
rs6790988
rs75664430
rs12932179
rs13062095
rs28502153
rs11426156
rs142426306
rs34550383
rs202034370
rs11212631
rs11412296
rs28363070
rs185174247
rs611646
rs1985369
rs35446936
rs8102497
rs79977579
rs4724
rs6669563
rs137901416
rs3785074
rs117034449
rs10112752
rs932002
rs80324517
rs78491606
rs6776756
rs6587577
rs7790856
rs73730598
rs1611236
rs117512405
rs61748181
rs111950327
rs35640778
rs3891167
rs59409453
rs9600019
rs1332941
rs9955360
rs28577594
rs143190905
rs11557154
rs16978028
rs10768683
rs7705526
rs11699829
rs10905255
rs2555104
rs116863223
rs188918174
rs5742915
rs112394943
rs66731853
rs4616688
rs2276182
rs56799554
rs2967355
rs76065543
rs17803849
rs144204502
rs1291143
rs8105767
rs11117354
rs139795227
rs41304832
rs11991877
rs3767952
rs12451892
rs939916
rs131797
rs10774624
rs12412214
rs6881568
rs4498805
rs6584579
rs12925933
rs3093888
rs142730696
rs4695407
rs55747751
rs111527438
rs4530278
rs41269079
rs77231040
rs2538745
rs2230590
rs7099229
rs6054257
rs762679
rs80116508
rs9940099
rs7209057
rs869785
rs7164950
rs76219171
rs6590343
rs1003322
rs2977608
rs6007020
rs117630647
rs73581419
rs45604339
rs77732866
rs56061761
All − MR Egger
All − Inverse variance weighted
−2
−1
0
1
2
3
MR effect size for
'telomere length || id:ieu−b−4879' on 'IDP_dMRI_TBSS_L1_Uncinate_fasciculus_L |
